# Effects of cottonseed meal on growth performance, liver redox status, and serum biochemical parameters in goslings at 1 to 28 days of age

**DOI:** 10.1186/s12917-022-03438-7

**Published:** 2022-09-15

**Authors:** Jun Yu, Zhengfeng Yang, Haiming Yang, Zhiyue Wang

**Affiliations:** 1 College of Animal Science and Technology, Yangzhou University, Yangzhou, 225009 Jiangsu Province China; 2 Joint International Research Laboratory of Agriculture and Agri-Product Safety of Ministry of Education of China, Yangzhou University, Yangzhou, 225009 Jiangsu Province China

**Keywords:** Cottonseed meal, Gossypol, Growth performance, Serum biochemical parameters, Antioxidant capacity, Goose

## Abstract

**Background:**

Cottonseed meal (CSM), a relatively rich source of protein and amino acids, is used as an inexpensive alternative to soybean meal (SBM) in poultry diets. However, the toxicity of free gossypol in CSM has been a primary concern. The present study was conducted to investigate the effects of CSM on growth performance, serum biochemical parameters, and liver redox status in goslings at 1 to 28 days of age. Three hundred 1-day-old male goslings were randomly divided into 5 groups (10 goslings/pen, 6 replicate pens/group) and subjected to a 28-day experiment. Five isonitrogenous and isoenergetic diets were formulated such that 0% (control), 25% (CSM_25_), 50% (CSM_50_), 75% (CSM_75_), and 100% (CSM_100_) of protein from SBM was replaced by protein from CSM. The free gossypol contents in the five diets were 0, 56, 109, 166, and 222 mg/kg, respectively.

**Results:**

The results showed that dietary CSM was associated with linear decreases in body weight, average daily feed intake and average daily gain and linear increases in the feed-to-gain ratio from 1 to 28 days of age (*P* < 0.001). As the dietary CSM concentration increased, a numerical increase was found in the mortality of goslings. According to a single-slope broken-line model, the breakpoints for the average daily gain of dietary free gossypol concentration on days 1 to 14, 15 to 28, and 1 to 28 occurred at 23.63, 14.78, and 18.53 mg/kg, respectively. As the dietary CSM concentration increased, serum albumin (*P* < 0.001) concentrations decreased linearly and serum uric acid (*P* = 0.011) increased linearly. The hydroxyl radical scavenging ability (*P* = 0.002) and catalase (*P* < 0.001) and glutathione peroxidase (*P* = 0.001) activities of the liver decreased linearly with increasing dietary CSM. However, dietary CSM did not affect the concentrations of reactive oxygen metabolites, malondialdehyde, or protein carbonyl in the liver.

**Conclusions:**

The increasing dietary CSM increased the concentration of free gossypol and altered the composition of some amino acids in the diet. A high concentration of CSM reduced the growth performance of goslings aged 1 to 28 days by decreasing feed intake, liver metabolism, and antioxidant capacity. From the primary concern of free gossypol in CSM, the tolerance of goslings to free gossypol from CSM is low, and the toxicity of free gossypol has a cumulative effect over time.

## Background

Feed cost accounts for approximately 70% of the total cost of poultry production [1], and the protein source accounts for a large proportion of that cost. Therefore, some less expensive byproducts that can replace soybean meal (SBM) as a protein source are used in goose feed by diet formulators and poultry producers in China [2–4].

Cottonseed meal (CSM), an oil industry byproduct, is an inexpensive alternative to SBM in poultry diets due to its relatively high concentrations of protein (30 to 50%) and amino acids [5]. However, the toxicity of free gossypol in CSM has been a primary concern, limiting its application as a raw protein source in poultry feed [6]. Free gossypol, a polyphenolic compound, is associated with reduced body weight (BW) gain, feed intake, increased mortality, and decreased blood hemoglobin and serum total protein in broilers or meat ducks [7–11]. However, the adverse effects of CSM on poultry were not observed in several trials [4, 12, 13]. The tolerance level of birds to free gossypol is affected by many factors, including the source and concentration of CSM, strain and age of birds, protein content and quality, feeding duration, and dietary iron and lysine [6]. Currently, many studies have been conducted on the response of chickens and meat ducks to free gossypol from CSM [7–11]. Swiatkiewicz et al. [5] reviewed the literature on CSM use in poultry and reported that CSM is an acceptable ingredient in poultry diets and can be safely consumed at a 10 to 15% dietary level, i.e., below 100 mg/kg free gossypol in the diet, partially replacing SBM. The tolerance level of goslings to free gossypol in the diet has not been well studied and evaluated.

In poultry species, gossypol is considered a hepatic toxin. The development of perivascular lymphatic aggregation, biliary hyperplasia, and hepatic cholestasis are typical syndromes of gossypol toxicity in chickens [7]. A study on meat ducks also showed that liver damage increased with increased free gossypol concentrations from CSM [9]. These liver injuries are partly due to the formation of free radicals since gossypol plays a major role in forming reactive oxygen species (ROS) through redox cycling by electron transfer functions [14]. Further research is needed to clarify the effect of gossypol on the redox state of goose liver.

The hypothesis in this study is that geese could tolerate a certain concentration of free gossypol from CSM, and a high concentration of CSM in the diet would adversely affect goose growth performance and alter the liver’s redox state. Therefore, the present study aimed to evaluate the effects of SBM replacement with CSM on the growth performance, serum biochemical parameters, and liver redox status in goslings at 1 to 28 days of age, investigating the potential harm of the presence of free gossypol in CSM.

## Methods

### Animals, experimental design, and management

This experiment was performed at Yangzhou University Experimental Farm (Gaoyou, China). A total of 300 1-d-old male Jiangnan White goslings (90.35 ± 4.5 g) were obtained from a commercial hatchery (Jiangsu Lihua Animal Husbandry Co. Ltd., Changzhou, China). The goslings were randomly divided into 5 dietary treatments with 6 replicate pens per treatment and 10 goslings per pen, according to a completely randomized design. The test period lasted for 28 d. Five isonitrogenous and isoenergetic experimental diets were formulated to meet or exceed the nutrient requirements of geese according to the NRC [15] and previous studies on medium-sized geese from our laboratory [16, 17]. A corn-soybean meal basal diet (as-fed basis) was used as the control; 25, 50, 75 or 100% of dietary protein provided by SBM was replaced with CSM in the other 4 diet groups (7.08, 14.15, 21.23 and 28.30% CSM, which are referred to as the CSM_25_, CSM_50_, CSM_75_, and CSM_100_ groups, respectively). All feed ingredients were ground in a hammer mill and passed through a 2-mm screen before mixing. The feed was given to the geese in mash form. The contents of free gossypol in CSM and experimental diets were determined by HPLC according to Zeng et al. [10]. The CSM contained 780 mg free gossypol/kg. The compositions of the experimental diets are listed in Table 1.

**Table 1:** Feed ingredient and nutrient composition of experimental diets for d 1 to 28

Items	Control^a^	CSM_25_	CSM_50_	CSM_75_	CSM_100_
Ingredient (%, as-fed basis)					
Corn	59.50	61.02	62.55	64.07	65.60
Soybean meal (46.96% crude protein)	27.60	20.70	13.80	6.90	0.00
Cottonseed meal (46.14% crude protein)	0.00	7.08	14.15	21.23	28.30
Rice husk	3.90	3.17	2.45	1.72	1.00
Wheat bran	5.40	4.36	3.31	2.27	1.23
Limestone	1.00	1.07	1.15	1.23	1.30
Calcium hydrogen phosphate	1.16	1.12	1.08	1.04	1.00
DL-Methionine	0.14	0.14	0.13	0.13	0.12
L-lysine HCl	0.00	0.04	0.08	0.11	0.15
Salt	0.30	0.30	0.30	0.30	0.30
Premix^b^	1.00	1.00	1.00	1.00	1.00
Total	100.00	100.00	100.00	100.00	100.00
Metabolizable energy^c^ (MJ/kg, as-fed basis)	11.39	11.39	11.39	11.39	11.39
Dry matter^d^ (DM)	87.74	87.84	87.78	87.82	87.86
Nutrient level (%, DM basis)					
Crude protein^d^	21.82	21.84	21.76	21.81	21.85
Crude fiber^d^	6.10	6.20	6.06	6.13	6.15
Calcium^d^	0.90	0.89	0.89	0.89	0.89
Available phosphorus^c^	0.43	0.43	0.43	0.43	0.43
Lysine^d^	1.06	1.05	1.05	1.04	1.04
Methionine^d^	0.44	0.42	0.44	0.43	0.44
Arginine^d^	1.24	1.35	1.54	1.64	1.78
Threonine^d^	0.71	0.71	0.69	0.68	0.69
Histidine^d^	0.44	0.43	0.46	0.44	0.46
Isoleucine^d^	0.80	0.77	0.74	0.69	0.66
Leucine^d^	1.53	1.48	1.45	1.39	1.26
Phenylalanine^d^	0.95	0.97	0.96	0.96	1.01
Valine^d^	0.81	0.82	0.80	0.81	0.81
Free gossypol^d^ (mg/kg, as-fed basis)	0	56	109	166	222

^a^Control group was fed a corn-soybean meal basal diet; CSM, cottonseed meal; CSM_25_, CSM_50_, CSM_75_, and CSM_100_ indicate that 25, 50, 75, and 100% of the protein content provided by soybean meal in the control diet was replaced with CSM respectively

^b^Provided per kilogram of complete diet: Vitamin A, 9000 IU; vitamin D, 3000 IU; vitamin E, 18 IU; vitamin K, 1.5 mg; vitamin B_1_, 0.9 mg; vitamin B_2_, 8 mg; vitamin B_6_, 3.2 mg; vitamin B_12_, 0.01 mg; nicotinic acid, 45 mg; pantothenic acid, 11 mg; folic acid, 0.65 mg; biotin 0.05 mg; choline, 0.35 g; Fe (as ferrous sulphate), 60 mg; Cu (as copper sulphate), 10 mg; Mn (as manganese sulphate), 95 mg; Zn (as zinc sulphate), 90 mg; I (as potassium iodide), 0.5 mg; Se (as sodium selenite), 0.3 mg

^c^Calculated values

^d^Analyzed values

The geese were housed in wire-floor pens (1.5 m × 0.9 m) and had ad libitum access to feed and water. Each pen was equipped with a separate feeder and 4 automatic nipple drinkers. The geese were exposed to a 24-h photoperiod from d 1 to 14 and an 18-h photoperiod from d 15 to 28. The house temperature was maintained at 28 to 30℃ for the first week and decreased by 2℃ each week until the house temperature was approximately 22℃ on d 28. This study was carried out in compliance with the ARRIVE guidelines. The experimental protocol and use of animals were approved by the animal care and use committee of Yangzhou University (Yangzhou, China).

### Sample collection and preparations

Birds were individually weighed at arrival. On d 14 and 28, after a 6-h feed withdrawal, goslings were weighed, and feed consumption was recorded by pen. The average daily feed intake (ADFI), average daily gain (ADG), and feed-to-gain ratio (F:G) were calculated. Mortality and the BW of the dead goose were recorded when death occurred. At the end of the trial, 30 goslings (6 goslings/treatment) with average BW were selected. Approximately 2 mL of blood was collected via the wing vein in a glass plain tube (no additive) and centrifuged at 2,000 × *g* for 10 min at 4 °C. Serum was collected and stored at –20 °C until biochemical parameter analysis. Once the blood was collected, birds were slaughtered by exsanguination. Samples of the liver (left lobes) were excised, flash-frozen in liquid nitrogen, and stored at –70 °C until further analysis.

### Sample analyses

Serum concentrations of alanine aminotransferase (ALT), aspartate aminotransferase (AST), total protein (TP), albumin (ALB), globulin (GLOB), and uric acid (UA) were determined using a UniCel DxC 800 Synchron fully automatic biochemical analysis system (Beckman Coulter, Los Angeles, CA, USA).

The liver samples were homogenized according to a previously described method [18]. The protein concentrations of the liver homogenates were determined using a Total Protein Quantitative Assay Kit (Nanjing Jiancheng Bioengineering Institute, Nanjing, China). The antioxidant capacity of the liver was evaluated in terms of the scavenging abilities of superoxide radical (O_2_•¯) and hydroxyl radical (OH•), the activities of superoxide dismutase (SOD) and catalase (CAT), glutathione (GSH) concentration, and the activities of GSH-related enzymes. These indicators were determined using a 725 N ultraviolet–visible spectrophotometer (INESA Scientific Instrument Co., Ltd., Shanghai, China) with respective commercial kits (Nanjing Jiancheng Bioengineering Institute, Nanjing, China). The results were calculated based on the protein concentrations of liver homogenates and are expressed as units per milligram of protein for O_2_•¯ scavenging ability (U/mg protein) and units per gram of protein for OH• scavenging ability (U/g protein). The activities of SOD and CAT are expressed as units per milligram of protein (U/mg protein). The GSH concentration is expressed as milligrams per gram of protein (mg/g protein). The activities of glutathione reductase (GR), glutathione peroxidase (GSH-Px), and glutathione S-transferase (GST) were calculated based on the protein concentration and are expressed as units per milligram of protein (U/mg protein).

The reactive oxygen metabolites (ROM) concentrations in the liver were measured spectrophotometrically according to the d-ROM test based on Costantini and Dell’Omo [19]. The results are expressed as millimoles of hydrogen peroxide (H_2_O_2_) per gram of protein in the liver (mmol H_2_O_2_/g protein). The concentration of malondialdehyde (MDA) was evaluated by the thiobarbituric acid reactive substance reaction. The MDA assay kit was purchased from Nanjing Jiancheng Bioengineering Research Institute (Nanjing, China), and the operation method followed the manufacturer’s instructions. The MDA concentration was calculated as nanomoles per milligram of protein (nmol/mg protein). The protein carbonyl (PC) concentrations were determined by the 2,4-dinitrophenylhydrazine reaction according to the method of Gaona-Gaona et al. [20] and are expressed as nanomoles per milligram of protein (nmol/mg protein) in the liver.

### Statistical analysis

Data were subjected to statistical analysis using the MIXED procedure of SAS software (SAS Institute Inc., Cary, NC). Polynomial contrasts were used to test the linear and quadratic responses to increasing dietary CSM concentrations. A pen (replicate) was used as an experimental unit for growth performance and a bird was used as an experimental unit for other parameters. All models included the fixed effect of dietary CSM concentration and random error of each observation. Mortality data were arcsin transformed before analysis. Data are expressed as the means and standard errors of the means. Differences were considered significant at *P* < 0.05, and tendency when 0.05 ≤ *P* < 0.10. In addition, broken-line regression analysis [21] was used to estimate the maximum concentration of dietary free gossypol concentration using the nonlinear regression procedure of SAS (SAS Institute Inc., Cary, NC). The broken-line model design is indicated by Y = L + U*(R − X), where Y = ADG, X = dietary free gossypol concentration (mg/kg), R = breakpoint (maximum limit), L = the response at X = R, and U = the slope of the curve. In this model, Y = L, when X < R.

## Results

### Growth performance

The BW, ADFI, ADG, F:G, and mortality of goslings fed diets with increasing dietary CSM concentrations are presented in Table 2. Increasing dietary CSM was associated with linear decreases in BW, ADFI, and ADG (*P* < 0.001). The F:G of goslings from 1 to 14 d increased as linear (*P* < 0.001) and quadratic (*P* = 0.034) response to increasing dietary CSM. The F:G of goslings from 15 to 28 d tended to increase (*P* = 0.065) as a linear (*P* = 0.044) response to increasing dietary CSM. The F:G of goslings from 1 to 28 d increased as a linear (*P* < 0.001) response to increasing dietary CSM. As the dietary CSM concentration increased, a numerical increase was found in the mortality of goslings.

**Table 2:** Effects of cottonseed meal on growth performance of goslings from 1 to 28 d of age

Item	Control^a^	CSM_25_	CSM_50_	CSM_75_	CSM_100_	SEM	*P-*value		
							Treatment	Linear	Quadratic
BW (g)									
Day 1	90.33	90.08	90.33	90.50	90.50	0.076	0.426	0.177	0.520
Day 14	554.22	518.43	456.73	441.15	341.22	15.180	< 0.001	< 0.001	0.191
Day 28	1379.49	1299.36	1101.38	1126.44	861.46	36.953	< 0.001	< 0.001	0.311
Days 1–14									
ADFI (g/d per bird)	60.65	56.86	49.87	50.27	39.70	1.543	< 0.001	< 0.001	0.390
ADG (g/d per bird)	33.13	30.60	26.18	25.07	17.90	1.084	< 0.001	< 0.001	0.181
F:G (g feed intake/g BW gain)	1.83	1.87	1.92	2.01	2.21	0.032	< 0.001	< 0.001	0.034
Mortality (%)	1.67	3.33	5.00	5.00	5.00	1.406	0.936	0.437	0.692
Days 15–28									
ADFI (g/d per bird)	137.7	128.5	115.0	118.5	91.2	3.549	< 0.001	< 0.001	0.304
ADG (g/d per bird)	58.95	55.78	46.05	48.95	37.16	1.666	< 0.001	< 0.001	0.561
F:G (g feed intake/g BW gain)	2.34	2.30	2.50	2.43	2.46	0.026	0.075	0.044	0.494
Mortality (%)	3.33	5.00	6.67	6.67	18.33	1.942	0.101	0.020	0.235
Days 1–28									
ADFI (g/d per bird)	99.03	91.98	81.81	84.08	65.12	2.444	< 0.001	< 0.001	0.311
ADG (g/d per bird)	46.04	43.19	36.11	36.99	27.53	1.317	< 0.001	< 0.001	0.310
F:G (g feed intake/g BW gain)	2.15	2.13	2.27	2.27	2.37	0.021	< 0.001	< 0.001	0.425
Mortality (%)	5.00	8.33	11.67	11.67	23.33	2.510	0.192	0.027	0.513

^a^Control group was fed a corn-soybean meal basal diet; CSM, cottonseed meal; CSM_25_, CSM_50_, CSM_75_, and CSM_100_ indicate that 25, 50, 75, and 100% of the protein content provided by soybean meal in the control diet was replaced with CSM respectively

*BW* Body weight, *ADFI* Average daily feed intake, *ADG* Average daily gain, *F:G* Feed to gain ratio, *SEM* Standard errors of the mean

**Fig. 1:**
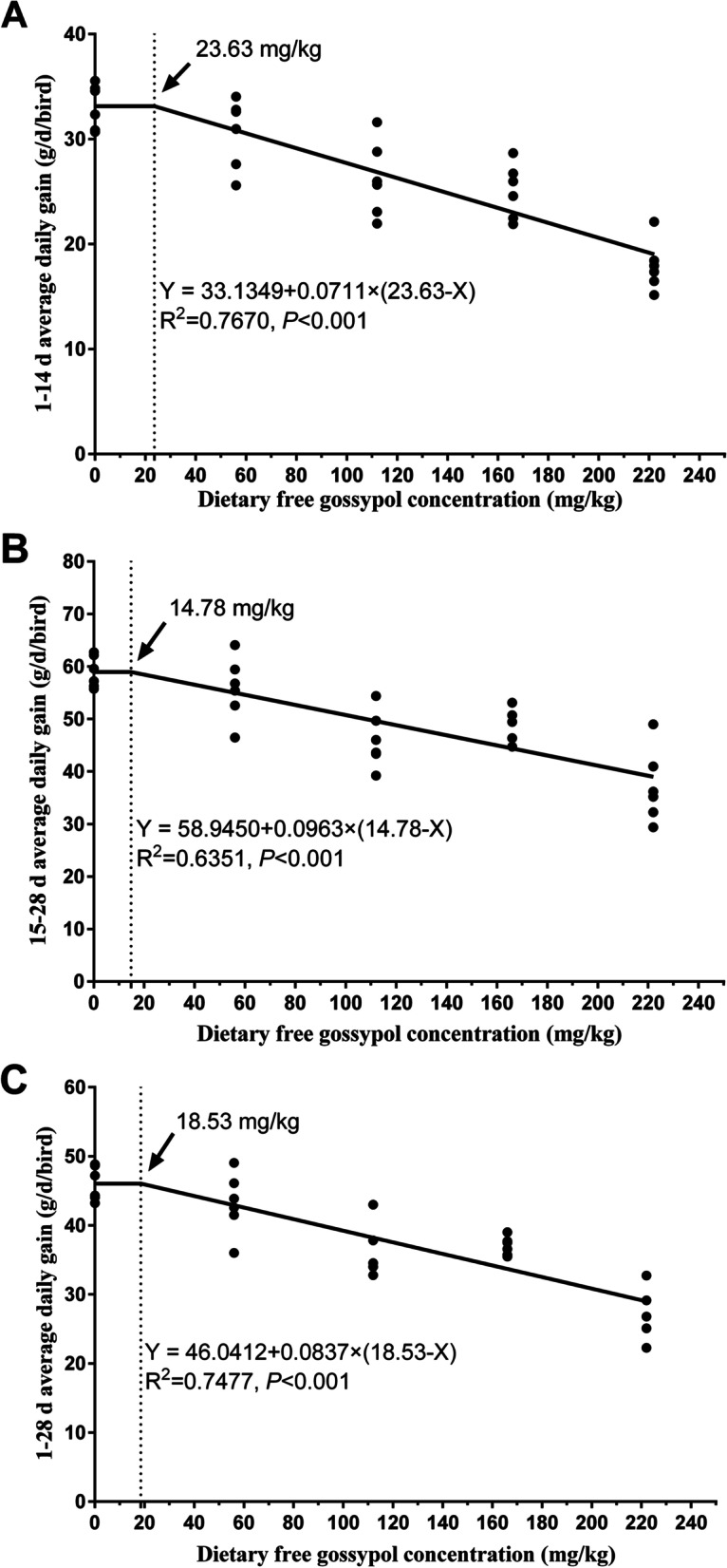
A broken-line analysis of average daily gain on goslings with increasing dietary free gossypol. Based on single-slope broken-line model, the breakpoints of dietary free gossypol concentration on day 1 to 14 (**A**), 15 to 28 (**B**), and 1 to 28 (**C**) occurred at 23.63, 14.78, and 18.53 mg/kg, respectively. The corresponding equations are: **A** Y = 33.1349 + 0.0711 × (23.63-X), breakpoint (BP) = 23.63 mg/kg (d 1 to 14); **B** Y = 58.9450 + 0.0963 × (14.78-X), BP = 14.78 mg/kg (d 15 to 28); **C** Y = 46.0412 + 0.0837 × (18.53-X), BP = 18.53 mg/kg (d 1 to 28). If dietary free gossypol concentration is less than BP, then (BP-X) = 0; if dietary free gossypol concentration is greater than BP, then (BP-X) = BP – dietary free gossypol concentration

The concentrations of free gossypol in diets increased with increasing dietary CSM (Table 1). The regression equation and maximum limits using single-slope broken-line analyses of dietary free gossypol concentration on ADG are presented in Fig. 1. The breakpoints for ADG of dietary free gossypol concentration on d 1 to 14, d 15 to 28, and d 1 to 28 occurred at 23.63, 14.78, and 18.53 mg/kg, respectively. The corresponding equations are Y = 33.1349 + 0.0711 × (23.63-X), *P* < 0.001, Fig. 1A, d 1 to 14; Y = 58.9450 + 0.0963 × (14.78-X), *P* < 0.001, Fig. 1B, d 15 to 28; Y = 46.0412 + 0.0837 × (18.53-X), *P* < 0.001, Fig. 1C, d 1 to 28.

### Serum biochemical parameters

As shown in Table 3, dietary CSM was associated with linearly decreasing concentrations of serum ALB (*P* < 0.001) and linearly increasing concentrations of serum UA (*P* = 0.011). As the dietary CSM concentration increased, the concentration of serum TP tended to decrease (*P* = 0.065) and in a linear response (*P* = 0.005).

**Table 3:** Effect of cottonseed meal on serum biochemical parameters of goslings at 28 d of age

Item	Control^a^	CSM_25_	CSM_50_	CSM_75_	CSM_100_	SEM	*P-*value		
							Treatment	Linear	Quadratic
Alanine aminotransferase (U/L)	39.50	27.80	34.50	44.00	41.17	2.868	0.473	0.378	0.424
Aspartate aminotransferase (U/L)	91.67	76.50	84.60	89.40	109.00	7.740	0.784	0.435	0.332
Total protein (g/L)	38.80	38.48	33.94	32.25	29.83	1.231	0.065	0.005	0.860
Albumin (g/L)	12.07	11.05	9.22	9.80	7.30	0.450	0.003	< 0.001	0.782
Globulin (g/L)	26.73	27.43	24.72	22.45	22.53	0.924	0.278	0.043	0.912
Uric acid (mmol/L)	131.83	147.67	129.50	159.16	223.33	11.274	0.041	0.011	0.097

^a^Control group was fed a corn-soybean meal basal diet; CSM, cottonseed meal; CSM_25_, CSM_50_, CSM_75_, and CSM_100_ indicate that 25, 50, 75, and 100% of the protein content provided by soybean meal in the control diet was replaced with CSM respectively. *SEM* Standard errors of the mean

### Antioxidant capacity in the liver

The effects of dietary CSM on the antioxidant capacity of the liver are shown in Table 4. The OH• scavenging ability (*P* = 0.016) and CAT (*P* = 0.002) and GSH-Px (*P* = 0.001) activities of the liver decreased linearly in response to increasing dietary CSM.

**Table 4:** Effects of cottonseed meal on antioxidant capacity in the liver of goslings at 28 d of age

Item	Control^a^	CSM_25_	CSM_50_	CSM_75_	CSM_100_	SEM	*P-*value		
							Treatment	Linear	Quadratic
Superoxide radical (U/mg protein)	1456.05	1374.75	1373.94	1382.44	1347.38	24.111	0.709	0.245	0.630
Hydroxyl radical (U/g protein)	184.25	151.10	126.79	134.94	116.42	7.182	0.016	0.002	0.239
Superoxide dismutase (U/mg protein)	236.83	231.67	232.23	227.85	213.66	2.990	0.126	0.018	0.336
Catalase (U/mg protein)	33.54	27.91	26.82	20.23	17.75	1.548	0.003	< 0.001	0.939
Reduced glutathione (mg/g protein)	67.45	62.98	65.64	69.41	62.22	4.271	0.986	0.902	0.911
Glutathione reductase (U/mg protein)	7.31	6.86	5.76	6.61	6.85	0.562	0.945	0.782	0.512
Glutathione peroxidase (U/mg protein)	205.35	177.87	186.00	186.84	165.64	3.497	0.002	0.001	0.912
Glutathione S-transferase (U/mg protein)	50.65	50.84	53.84	56.19	52.77	1.089	0.495	0.229	0.400

^a^Control group was fed a corn-soybean meal basal diet; CSM, cottonseed meal; CSM_25_, CSM_50_, CSM_75_, and CSM_100_ indicate that 25, 50, 75, and 100% of the protein content provided by soybean meal in the control diet was replaced with CSM respectively. *SEM* Standard errors of the mean

### Reactive oxygen metabolite, malondialdehyde and protein carbonyl concentrations in the liver

As shown in Table 5, dietary CSM did not affect the concentrations of ROM, MDA, or PC in the liver.

**Table 5:** Effects of cottonseed meal on the reactive oxygen metabolites, malondialdehyde, and protein carbonyl concentrations in the liver of goslings at 28 d of age

Item	Control^a^	CSM_25_	CSM_50_	CSM_75_	CSM_100_	SEM	*P-*value		
							Treatment	Linear	Quadratic
Reactive oxygen metabolites (mmol H_2_O_2_/g protein)	50.14	49.17	55.18	58.99	51.23	1.524	0.222	0.260	0.212
Malondialdehyde (nmol/mg protein)	0.63	0.56	0.76	0.60	0.60	0.048	0.776	0.983	0.634
Protein carbonyl (nmol/mg protein)	1.84	2.05	1.83	1.96	1.73	0.124	0.945	0.727	0.631

^a^Control group was fed a corn-soybean meal basal diet; CSM, cottonseed meal; CSM_25_, CSM_50_, CSM_75_, and CSM_100_ indicate that 25, 50, 75, and 100% of the protein content provided by soybean meal in the control diet was replaced with CSM respectively. *SEM* Standard errors of the mean

## Discussion

To reduce production costs, some inexpensive local crop byproducts have been used in goose diets in China because of the strong tolerance and adaptability to roughage in geese [2–4]. Cottonseed meal can be used as an inexpensive partial replacement for SBM in poultry diets. In practice, the quantity of CSM that can be incorporated into the diet depends largely on the amount of gossypol in the meal [22]. In the present study, goslings fed diets containing CSM exhibited linearly reduced ADFI and ADG and linearly increased F:G during the entire or part of the trial period. Similar results in broilers were also reported by Henry et al. [7], who found that chicks fed gossypol at 400 mg/kg of feed had a poor feed conversion ratio compared with the other treatment. Zeng et al. [10] reported that ducks fed a diet containing 33.11% CSM (152.9 mg free gossypol/kg) had reduced ADFI and ADG from d 1 to 14. The reduction in BW gain and feed intake are common signs of gossypol toxicosis [7, 23]. Differences in tolerance to free gossypol on individual geese caused considerable differences in mortality in replicate pens in the same treatment. Therefore, there was no statistically significant difference in mortality between treatments. However, increasing dietary CSM concentrations resulted in a big numerical increase in mortality. The effect of CSM on the mortality of goslings should be of concern.

The application of CSM caused an imbalance in the amino acid composition of the diet. The concentration of lysine and methionine in the diet formulation had been considered, but it caused a decrease in leucine and isoleucine and an increase in arginine, which may have potential adverse effects. Branched-chain amino acids (leucine, isoleucine, and valine) are among the key regulators of protein synthesis, and their optimal ratio is essential to induce nutrient sensors to signal myocyte proliferation and differentiation leading to muscle growth and development [24]. Gradual increases in dietary arginine may have dual positive and negative effects, as slightly higher dietary arginine levels promoted growth, while excessive dietary arginine levels exhibited reduced growth [25]. Lysine and arginine compete for intestinal and renal transporters, and excessive arginine reduces lysine utilization [26]. In addition, severe feather pecking was also observed in the CSM_100_ group (28.30% CSM), which may be a consequence of the imbalance in the amino acid composition of the diet. We also observed a reduction of approximately 30% in the ADFI of goslings fed the CSM_100_ diet, which may lead to protein, amino acid, and mineral deficiencies, increasing the risk of feather pecking [27].

In this report, we attempted to estimate the maximum limit of dietary free gossypol by broken-line regression analysis based on ADG and mortality. However, the fitting degree of the broken-line regression model based on mortality is low, so it is omitted. The concentration of free gossypol in the diet did not cause acute death of the goslings; therefore, the adverse effect of free gossypol on the goslings was firstly expressed by growth depression and eventually even death. Predictably, the maximum limit of dietary free gossypol on mortality was higher than that on ADG. Cravens et al. [28] reported that aflatoxin B1 concentrations that increased mortality were higher than those that decreased weight gain in young broilers.

In this study, the dietary free gossypol concentration breakpoint based on ADG was 23.63 mg/kg on days 1 to 14, 14.78 mg/kg on days 15 to 28, and 18.53 mg/kg on days 1 to 28. Contrary to what we expected, the maximum limit of free gossypol in older goslings was lower than that in younger goslings. This observation was inconsistent with the findings of Zeng et al. [10], who found that the tolerance of meat ducks aged 1 to 21 days to dietary free gossypol concentration increased with increases in duck age. This result may be attributed to several factors. Firstly, goslings are particularly sensitive to dietary free gossypol concentration. The maximum free gossypol concentration in broiler diets was estimated to be approximately 200 mg/kg [29, 30]. The dietary free gossypol concentration of meat ducks should be lower than 77 mg/kg from 1 to 21 days [10]. In this study, the maximum limit of dietary free gossypol concentration was lower in goslings of similar age than broilers or meat ducks. Furthermore, the negative impact of free gossypol is time-dependent. Free gossypol accumulated in animal tissue over time, and the adverse effects on growth performance could be due to the accumulation of free gossypol in the tissue [11]. The growth inhibition caused by free gossypol in goslings affects the growth of detoxification organs such as the liver, thus affecting the tolerance to dietary free gossypol at higher ages. This result was confirmed by the fact that more deaths occurred at 15 to 28 days rather than at 1 to 14 days. Generally, the dietary free gossypol concentration of goslings should be less than 18.53 mg/kg (equivalent to 2.38% CSM in this study) from 1 to 28 days.

To a certain extent, serum biochemical parameters can reflect the metabolic status of animals and the changes in some tissues and organs. In the present study, the serum concentrations of TP trended to decrease with increasing dietary CSM, which was mainly due to a significant decrease in serum ALB. It has been reported that gossypol is transported in the blood by binding to the high-affinity binding site of serum ALB [31]. The decrease in serum protein concentration implies the disorder of protein synthesis and metabolism. This observation is consistent with that of Zeng et al. [9], who reported that the serum TP, ALB, and GLOB contents linearly decreased with increasing dietary CSM at 35 d of age in meat ducks. Uric acid is the final product of protein catabolism in poultry. In this study, a linear increase in serum UA content was associated with a linear decrease in BW, serum TP and ALB, indicating that the utilization of protein decreased with increasing dietary CSM concentrations. These results were confirmed by Zhu et al. [11], who found that the serum urea nitrogen concentration in ducklings fed diets with 80, 160, and 240 mg free gossypol/kg was higher than that in birds fed the control diet at d 21.

The liver may be one of the first tissues negatively affected by dietary gossypol because the liver has the highest concentration of gossypol residues among all the tissues [9, 32]. The possible mechanism of gossypol’s effect on the liver is partly due to its involvement in forming free radicals [14], which alter the liver’s redox state. Gossypol can combine with iron, thereby inhibiting the synthesis of respiratory enzymes and generating excessive ROS in mitochondria [33]. Gossypol greatly accelerates the production of OH• from hydrogen peroxide by up to eightfold in the presence of Fe3 +—EDTA [34]. In this experiment, we determined the O_2_•¯ and OH• scavenging abilities, SOD and CAT activities, GSH content, and GSH-related enzyme activities (including GR, GSH-Px, and GST) to evaluate the antioxidant capacity of the liver. The O_2_•¯and OH• are two representative ROS. When ROS levels exceed the scavenging capability of the antioxidant system, oxidative stress occurs [35]. In the present study, the OH• scavenging ability of the liver linearly decreased in response to the increase in dietary CSM. Hydrogen peroxide may be converted into water by the enzymes CAT and GSH-Px [36]. In this study, the linear decreases in CAT and GSH-Px activities in the liver demonstrated that gossypol reduced the antioxidant capacity in goslings. The data showed that a high concentration of CSM in the feed suppressed antioxidant activity in the goose liver.

The ROM compounds are generated by the reaction of ROS with biomacromolecules [19]. Disruption of the intracellular redox balance leads to a state of oxidative stress, during which proteins, nucleic acids, lipids, and other macromolecules can suffer severe damage [37]. The MDA and PC concentrations represent the evaluation indicators of lipid peroxidation and protein oxidation by free radicals, respectively. Intriguingly, although the OH• scavenging ability and CAT and GSH-Px activities were decreased, dietary CSM concentrations had no effects on the ROM, MDA, and PC concentrations in geese livers on d 28. To maintain stability in ROM, MDA, and PC concentrations, goslings may have alleviated the negative effect of free gossypol on free radical balance via a voluntary reduction in feed intake.

## Conclusions

In conclusion, the increasing dietary CSM increased the concentration of free gossypol and altered the composition of some amino acids in the diet. A high concentration of CSM would have adverse effects on goslings from day 1 to 28, including poor growth performance and reduced liver metabolism and antioxidant capacity. From a single-slope broken-line analysis of free gossypol, the main concern in CSM, goslings could tolerate low concentrations of free gossypol (less than 18.53 mg/kg) from 1 to 28 days, and the toxicity of free gossypol has a cumulative effect over time.

## Data Availability

The datasets supporting the conclusions of this article are included within the article.

## Abbreviations

ADFI: Average daily feed intake
ADG: Average daily gain
ALB: Albumin
ALT: Alanine aminotransferase
AST: Aspartate aminotransferase
BW: Body weight
CAT: Catalase
CSM: Cottonseed meal
F:G: Feed to gain ratio
GLOB: Globulin
GR: Glutathione reductase
GSH: Reduced glutathione
GSH-Px: Glutathione peroxidase
GST: Glutathione S-transferase
MDA: Malondialdehyde
O_2_**·¯**: Superoxide radical
OH**·**: Hydroxyl radical
PC: Protein carbonyl
ROM: Reactive oxygen metabolites
SEM: Standard errors of the mean
SOD: Superoxide dismutase
TP: Total protein
UA: Uric acid
